# Leptospirosis Cases Infected with Uncommon Serogroups, Puerto Rico, 2013–2015

**DOI:** 10.4269/ajtmh.17-0538

**Published:** 2017-11-06

**Authors:** Hector Gorbea, Enid J. Garcia-Rivera, Hilda Torres, Olga D. Lorenzi, Aidsa Rivera, Renee L. Galloway, Tyler M. Sharp

**Affiliations:** 1 University of Puerto Rico School of Medicine, San Juan, Puerto Rico;; 2 Division of Vector-Borne Diseases, Centers for Disease Control and Prevention, San Juan, Puerto Rico;; 3 Division of High Consequence Pathogens and Pathology, Centers for Disease Control and Prevention, Atlanta, Georgia

## Abstract

Leptospirosis is an emerging bacterial zoonosis that is endemic but underrecognized throughout the tropics. Through prospective surveillance for acute febrile illness (AFI) among patients who presented to the emergency department of a hospital located in an urban region of Puerto Rico, four patients with laboratory-confirmed leptospirosis were identified. All patients had signs and symptoms of AFI, including fever, headache, and dehydration. Three patients had leukocytosis with thrombocytopenia and were admitted to the hospital. One hospitalized patient presented with jaundice, icteric sclera, and hematuria and developed rhabdomyolysis, whereas another patient with pulmonary edema was admitted to the intensive care unit. Microscopic agglutination titers among the four patients were highest against serogroups Icterohaemorrhagiae (serovar Mankarso), Australis (serovar Bratislava), Bataviae (serovar Bataviae), and Canicola (serovar Canicola). These case reports demonstrate that infection with these apparently uncommon serogroups can result in illness ranging from mild to life-threatening.

## INTRODUCTION

Leptospirosis is a bacterial zoonosis that is endemic throughout the tropics and is caused by infection with *Leptospira* species bacteria that are transmitted through direct or indirect contact with animal urine.^1^ Most patients infected with *Leptospira* spp. will experience either no symptoms of disease or a self-limited acute febrile illness (AFI).^1^ Among hospitalized leptospirosis patients, 5–15% die typically because of organ (e.g., kidney and liver) failure, pulmonary hemorrhage, and/or septic shock.^1,2^ Each year an estimated 1 million cases of leptospirosis and nearly 60,000 deaths^3^ result in a loss of 2.9 million disability-adjusted life years.^4^

The *Leptospira* genus comprises at least 13 pathogenic species, most of which belong to *Leptospira interrogans* and > 200 serovars which are arranged into 24 serogroups.^1,5^ Although the *Leptospira* genus was classically classified into serogroups according to antigenic determinants, more recent classifications are molecular.^1,5^ Serovars are often associated with particular animal reservoir hosts in which they may cause both disease and persistent infection resulting in long-term shedding of infectious leptospires.^1^ Disease severity in humans^6,7^ and animals^8^ may vary according to the infecting serovar.

Puerto Rico is a United States territory located in the Caribbean, and in 2015 had a population of 3.5 million residents.^9^ Although leptospirosis is a reportable disease, its clinical similarity with dengue,^10^ lack of timely diagnostic testing,^11^ and potentially suboptimal clinical awareness^11^ together result in underreporting of leptospirosis.^11–13^

Here, we describe four leptospirosis cases from Puerto Rico in patients who were presumptively infected with serogroups that have been infrequently identified as a cause of human disease.

## MATERIALS AND METHODS

Data were collected during July 29, 2013, through August 24, 2015, from the Sentinel Enhanced Dengue Surveillance System (SEDSS) site located at the University of Puerto Rico (UPR) Hospital, Carolina. Patients presenting to the emergency department (ED) with fever or a history of fever in the past 7 days were eligible for participation. Clinical specimens were collected upon enrollment and upon hospital discharge or follow-up visit, and diagnostic testing was performed as previously described.^14^ All patients enrolled in SEDSS provided informed consent. Additional data were collected through chart abstraction for leptospirosis patients. The Institutional Review Board of the UPR Medical Sciences Campus approved the protocols for SEDSS and this study.

Patients for whom both acute and convalescent serum specimens were available were tested for infection with *Leptospira* spp. by microscopic agglutination test (MAT) for 20 *Leptospira* reference antigens representing 17 serogroups.^15^ For cases with an MAT-positive convalescent specimen, the acute serum specimen was tested by MAT and polymerase chain reaction (PCR) targeting the *lipL32* gene.^16^ As insufficient DNA was available to perform multilocus sequence typing to identify infecting serovars, specimens that tested positive by PCR were further tested to identify pathogenic *Leptospira* species.^17^ Unless otherwise specified, all diagnostic testing performed under the SEDSS protocol for pathogens other than *Leptospira* spp. (i.e., dengue virus types 1–4, chikungunya virus, influenza A and B viruses, and 12 respiratory pathogens) was negative.

## CASE REPORTS

### Case 1.

In July 2013, a 25-year-old male presented to the ED with complaints of a 4-day history of fever, myalgia, bone pain, headache, malaise, chills, and nausea (Table 1). Two days earlier he had sought care at a local health center where he was found to be dehydrated and was given intravenous (IV) fluids. Upon presentation to the ED, he was afebrile, and laboratory results demonstrated thrombocytopenia and mildly elevated liver function tests (LFTs) (Tables 2 and 3). He was given a provisional diagnosis of viral syndrome with a rule-out of dengue, given acetaminophen and IV fluids, and admitted for care.

**Table 1:** Demographic and clinical characteristics of four leptospirosis cases from northeastern Puerto Rico, 2013–2015

Case #	Age (years)	Sex	Exposure history	Leptospirosis diagnostic testing					Discharge diagnosis
				Acute specimen			Convalescent specimen		
				DPO of collection	PCR result	MAT titer* (Serogroup)	DPO of collection	MAT titer* (Serogroup)	
1	25	M	Contact with stagnant water and unspecified animals	5	NP	100 (Mankarso)	8	3,200 (Mankarso)	Dengue
2	54	F	None reported	2	Pos	Neg	12	3,200 (Bratislava)	Pneumonia
3	27	M	Exposure to rats, and walking barefoot	3	Pos	Neg	7	1,600 (Bataviae)	Leptospirosis
4	22	M	Contact with stagnant water, exposure to rats, and household member with recent acute febrile illness	2	Neg	Neg	18	800 (Bataviae and Canicola)	Viral syndrome

DPO = day postillness onset; F = female; M = male; MAT = microscopic agglutination test; Neg = negative; NP = testing not performed; PCR = polymerase chain reaction; Pos = positive.

* Reciprocal titers are shown.

**Table 2:** Laboratory values of three leptospirosis patients, Puerto Rico, 2013–2015

Case ID	WBC				Platelets				Hematocrit			
	First		Highest		First		Lowest		First		Lowest	
	DPO	Value	DPO	Value	DPO	Value	DPO	Value	DPO	Value	DPO	Value
1	4	10.9	8	12.3	4	79	7	66	4	41.4	7	34.5
2	3	12.4	8	33.0	3	183	7	28	3	39.3	6	33.4
3	5	8.9	10	10.3	5	106	6	73	5	46.0	8	35.5

DPO = day postillness onset; WBC = white blood cell count. Units: WBC and platelets = ×10^3^/mm^3^; hematocrit = %.

**Table 3:** Laboratory values of three leptospirosis patients, Puerto Rico, 2013–2015

Case ID	Creatinine				Total Bilirubin				AST				ALT			
	First		Highest		First		Highest		First		Highest		First		Highest	
	DPO	Value	DPO	Value	DPO	Value	DPO	Value	DPO	Value	DPO	Value	DPO	Value	DPO	Value
1	4	1.2	NA	NA	4	1.3	NA	NA	4	92	NA	NA	4	136	NA	NA
2	3	3.8	3	3.8	3	1.3	3	1.3	3	37	6	1,807	3	43	6	696
3	5	1.0	7	3.4	5	6.5	8	20.1	6	138	7	163	6	93	7	143

ALT = alanine aminotransferase; AST = aspartate aminotransferase. Units: AST and ALT = international units/L; creatinine and total bilirubin = mg/dL.

On the first day of hospitalization, he remained afebrile and developed nausea, vomiting, and dry cough. His lungs were consistently clear to auscultation and chest X-rays were unremarkable. Repeat laboratory values on the third day of hospitalization demonstrated worsening thrombocytopenia. Additional laboratory results obtained the following day demonstrated leukocytosis and improving thrombocytopenia. He was discharged home on day 4 of hospitalization with a diagnosis of dengue.

A serum specimen collected on day 5 of illness was positive by MAT with a reciprocal titer of 100 against serogroup Icterohaemorrhagiae (serovar Mankarso). Serum collected upon discharge 8 days after illness onset had a reciprocal MAT titer of 3,200 against serovar Mankarso.

### Case 2.

A 54-year-old female presented to the ED in March 2014 with complaints of a 3-day history of fever, abdominal pain, vomiting, diarrhea, headache, and anorexia. On evaluation she had dry oral mucosa, and laboratory results demonstrated leukocytosis and elevated serum creatinine, amylase (130 units per liter [u/L]), and lipase (118 u/L). Ultrasound examination revealed evidence of cholelithiasis. The initial diagnostic impression was acute kidney injury with dehydration, IV piperacillin–tazobactam and IV fluids were initiated, and the patient was hospitalized.

On day 3 of hospitalization, the patient developed shortness of breath and had bibasilar crackles. Chest X-ray revealed bilateral confluent alveolar opacities. Echocardiogram demonstrated mild tricuspid and mitral valve regurgitation. Laboratory results demonstrated leukocytosis, a 15% drop in hematocrit, thrombocytopenia, and mildly elevated LFTs. Because of the onset of pulmonary edema, developing acute respiratory distress syndrome and concerns of respiratory tract infection, the patient was transferred to the intensive care unit and placed on oxygen supplementation. Furosemide was given, and antimicrobial therapy was changed to include azithromycin, imipenem, trimethoprine–sulfamethoxazole, linezolid, and oseltamivir. Laboratory results demonstrated worsening leukocytosis and thrombocytopenia and prominently elevated LFTs and lactate dehydrogenase (LDH) (9,976 u/L).

The following day she was feeling better, but with mild shortness of breath and continued bibasilar crackles. She continued to improve daily thereafter, was transferred to the regular ward on the seventh hospitalization day, and was discharged home on the 12th day of hospitalization.

All blood and sputum cultures were negative. A serum specimen drawn on the sixth day of symptoms (third day of hospitalization) was negative for *Leptospira* IgM antibody test performed at a commercial reference laboratory and also negative for mycoplasma IgM antibody and HIV. DNA specific to *L. interrogans* was detected by PCR in a serum specimen collected on day 2 of illness, which was negative by MAT. Serum collected upon patient discharge had a reciprocal MAT titer of 3,200 against serogroup Australis (serovar Bratislava).

### Case 3.

In March 2014, a 27-year-old male presented to the ED because of a 5-day history of back pain, chills, fever, headache, eye pain, and arthralgia. On physical examination, he had jaundice and icteric sclera, dry oral mucosa, and tender calves. Laboratory results revealed thrombocytopenia and elevated total bilirubin and creatinine phosphokinase (CPK; 1,572 u/L). Urinalysis revealed hematuria and bilirubinemia. He was diagnosed with suspected leptospirosis with rhabdomyolysis, hospitalized, and given IV ceftriaxone and fluid replacement.

Approximately 24 hours after arrival to the ED, the patient had worsening thrombocytopenia and elevated serum creatinine and LFTs. On the second day of hospitalization, he had vomiting but remained afebrile, serum creatinine and LFTs were further elevated, and CPK rose to 4,709 u/L. Ceftriaxone and fluid replacement were continued, and antiemetics were given. On day 3 of hospitalization, he remained afebrile, platelet count stabilized, and CPK decreased to 1,783 u/L. The patient’s symptoms improved and vomiting stopped, but jaundice and icteric sclera persisted through the following day. A renal sonogram on day 5 of hospitalization showed fluid along the right upper quadrant. He was discharged home on day 8 of hospitalization.

A leptospirosis IgM antibody test by a commercial reference laboratory with serum collected on the day of admission was negative. Tests for Hepatitis A, B, and C and blood cultures drawn on admission were negative. *Leptospira interrogans* DNA was detected by PCR in a serum specimen collected on day 3 of illness, which was negative by MAT. Serum collected upon patient discharge had a reciprocal MAT titer of 1,600 against serogroup Bataviae (serovar Bataviae).

### Case 4.

A 22-year-old male was evaluated at the ED on March 24, 2014, because of a 3-day history of fever, malaise, myalgia, nausea, rhinorrhea, sore throat, and headache. Physical examination revealed fever, underweight (body mass index = 18.1), and tachycardia. He was given acetaminophen and discharged from the ED with a diagnosis of viral syndrome.

Serum collected on day 2 of illness was negative by PCR and MAT. Convalescent serum collected 18 days after illness onset was positive by MAT with a reciprocal titer of 800 against serogroups Bataviae (serovar Bataviae) and Canicola (serovar Canicola) and was also positive for detection of anti-dengue virus IgM antibodies.

## DISCUSSION

Serologic evidence of infection with serogroups Icterohaemorrhagiae, Australis, Canicola, and Bataviae was found among patients with leptospirosis in Puerto Rico. Evidence of circulation of serovars from these serogroups has been infrequently documented in Puerto Rico, and most previously identified leptospirosis patients had serologic evidence of infection with Icterohaemorrhagiae.^12^ However, because serologic diagnostic testing may not accurately identify the infecting serogroup,^1^ we cannot rule out the possibility that patients may have been infected with a serogroup distinct from those identified by MAT. Nonetheless, because Icterohaemorrhagiae has been associated with increased likelihood of fatal outcome,^6,7^ this may suggest that other serogroups are comparatively less pathogenic in humans. Although Icterohaemorrhagiae is most frequently associated with exposure to the urine of rats, serogroup Bataviae is also associated with dogs, whereas Bratislava is associated with horses.^1^ This observation underscores the need for public awareness that not only rats but also many species of mammals can harbor infectious leptospires.

This case series demonstrates that the clinical severity of leptospirosis can range from mild to life-threatening. Early identification of leptospirosis patients is necessary to rapidly initiate antibiotic therapy, which has been associated with improved patient outcome.^18,19^ However, the overlapping clinical manifestations of leptospirosis, dengue, and other tropical AFIs create a diagnostic challenge. Because patients who develop life-threatening illness do not necessarily present for care with severe illness, there is also a need to identify early indicators of patients that will progress to having more severe disease. Such indicators at present are limited to clinical signs and symptoms and laboratory values^12,18^; however, the combination of clinical indicators with rapid diagnostic testing could prove to be instrumental in reducing morbidity and mortality attributable to leptospirosis.
